# Network meta-analysis of the efficacy of four traditional Chinese physical exercise therapies on the prevention of falls in the elderly

**DOI:** 10.3389/fpubh.2022.1096599

**Published:** 2023-01-04

**Authors:** Meichao Cheng, Ya Wang, Shun Wang, Wenxiao Cao, Xianliang Wang

**Affiliations:** ^1^School of Physical Education, Shandong University, Jinan, China; ^2^School of Physical Education, Huaibei Normal University, Huaibei, China

**Keywords:** Tai Chi, Ba Duan Jin, Yi Jin Jing, Wu Qin Xi, network meta-analysis

## Abstract

**Introduction:**

In recent years, traditional Chinese exercises have been passed down and reformed to play a significant role in the study of interventions for the treatment of falls in older people. However, few studies have evaluated the efficacy of various Chinese traditional exercises in the intervention of falls behavior in the elderly. In this study, four Chinese traditional exercises commonly used in clinical practice were selected as subjects to systematically evaluate the effectiveness of Tai Chi, Ba Duan Jin, Yi Jin Jing and Wu Qin Xi in intervening in the treatment of fall behavior in the elderly.

**Methods:**

We conducted a systematic review in accordance with the PRISMA guidelines. Four published randomized controlled trials (RCTs) of traditional Chinese exercise interventions for the treatment of falls behavior in older adults were searched through authoritative databases such as CNKI, Web of Science, PubMed, EMbase and the Cochrane Library, all from the time of construction to November 2022.

**Results:**

A total of 45 studies with 4 traditional interventions were included. Ba Duan Jin was more effective in improving TUGT [SMD = −1.93 (−2.49, −1.38), *P* < 0.05] and MFES [SMD = −33.45 (−63.93, −2.97), *P* < 0.05], while Yi Jin Jing was more effective in enhancing ECLSB [SMD = −0.19 (−5.12, −4.74), *P* < 0.05] and BBS [SMD = −5.79 (−10.80, −0.78), *P* < 0.05], both of which showed better effects.

**Discussion:**

The present evidence suggests that all four traditional Chinese body-building exercise therapies have a preventive effect on fall behavior in older adults. In clinical treatment and daily physical exercise, two exercises, BaDuan Jin and Yi Jin Jing, may be preferred to reduce the risk of falls in the elderly, but the exercise regimen of Qigong should be selected scientifically and rationally according to their actual conditions.

**Systematic review registration:**

https://www.crd.york.ac.uk/PROSPERO/#myprospero.

## 1. Introduction

According to the United Nations Development Programme, the population of older people aged 65 and above will increase to 340 million by 2040, and the increasing aging of the population will become a major social problem in China (1). Some studies have shown that ~20–35% of older adults aged 60 years and older experience fall behavior each year, and more than half of these falls result in injury, fracture, or even death (2). One study found that healthcare expenditures increase with the frequency of falls (3). Falling not only seriously affects the physical and mental health of the elderly but also brings a heavy burden to families and society, and if not taken seriously, the public health risks they pose will be incalculable.

A single-type movement and a more complete-type movement are currently the most common approaches to reducing the risk of falls among the aged population. The balance board, the Babbitt Ball, and walking are all examples of single-type movement (4). The clinical therapy impact favor the complete-type movement over the single-type movement (5). Traditional Chinese exercises have been widely adopted in the practice of fall behavior through inheritance and reform, and have shown promising clinical results. Tai Chi, Ba Duan Jin, Yi Jin Jing, Wu Qin Xi and other exercises have been demonstrated to increase muscle strength and balance function and significantly reduce the risk of falls in older adults (4, 5). The reason for this is that the traditional Chinese body-building exercises were founded on the idea of “Life as a whole” and integrate traditional Chinese medicine with traditional Chinese health care, which not only has positive effects on the physical and mental health of the elderly but also can effectively improve the muscle strength and balance of the elderly, thus strengthening the body and preventing falls.

In terms of the factors influencing fall behavior, the World Health Organization suggests that fall behavior is mainly formed by the interaction of social, environmental, biological and behavioral factors (6). Other studies have suggested that as many as 132 factors contribute to fall behavior (7); nonetheless, poor coordination, reduced muscle strength and unstable gait are the main causes. It has been proposed in other research that aspects outside exercise technique, such as exercise conditions, management strategies, guidance experience, interaction, and so on, contribute to the intervention effect of falls in the elderly. Therefore, the prevention and improvement of fall behavior is a systematic project that needs to take into account the influence of a combination of social, environmental and management factors, based on the scientific choice of exercise modality.

In summary, long-term and regular physical exercise is undoubtedly an effective measure to reduce the risk of falls in the elderly (5, 6, 8, 9), but few studies have compared the efficacy of a variety of traditional Chinese exercises, posing a problem for the choice of modality for patients. Consequently, this study employed network Meta-analysis to integrate the relevant clinical trial data and assess the evidence-based intervention measures, with the research object being four classic body-building exercises routinely used in clinical practice. Sorting the degree of success based on the primary outcome allows for the elimination of less effective strategies. The study aims to provide a reliable reference for the prevention and improvement of older people's behavior.

## 2. Materials and methods

The literature search strategy follows the PRISMA guidelines and the PICO framework. PRISMA is used as the basis for reporting systematic reviews with objectives. In addition, we have incorporated the participants, interventions, comparators, outcomes, and study design (PICOS) framework, which can help develop search strategies with a range of key questions to effectively find high-quality evidence.

### 2.1. Participants

(1) Age ≥ 60 years; (2) Physically fit and physically active, able to participate in exercise; (3) Sign the informed consent form and voluntarily participate in this study.

### 2.2. Interventions

Four Traditional Chinese Physical Exercise Therapies (Tai Chi, Ba Duan Jin, Yi Jin Jing and Wu Qin Xi).

### 2.3. Comparators

No traditional Chinese exercise training and a routine lifestyle or daily care approach. Routine life means that the subject does not participate in special exercise training and there are no exercise interventions. Daily care means that the subject undergoes regular rehabilitation therapy such as balance training, acupuncture, massage, etc.

### 2.4. Outcomes

Four measures of TUGT, ECLSB, BBS, and MFES.

### 2.5. Study design

(1) Determining the purpose and selection of the study and comparing the effects of four traditional Chinese exercises on older people's fall behavior; (2) Literature screening, 45 RCT studies involving 3,695 subjects were finally included; (3) Quality evaluation, the risk of bias assessment tool recommended by the Cochrance Collaboration Network was used to evaluate the quality of the literature; (4) Data analysis, R and ADDIS software were used to The different interventions were analyzed and the network relationships of the interventions were also mapped.

### 2.6. Inclusion and exclusion criteria

#### 2.6.1. Research type

Randomized controlled trials (RCTs) with no language or blinding constraints.

#### 2.6.2. Interventions

In accordance with the patients' actual conditions and the clear diagnostic criteria and therapeutic evaluation criteria established by the rehabilitation experts, those in the experimental group received Chinese traditional exercise therapy (Ba Duan Jin, Tai Chi, Wu Qin Xi, Yi Jin Jing), while those in the control group received routine life or daily nursing. Routine life means that the subject does not participate in special exercise training and there are no exercise interventions. Daily nursing means that the subject undergoes regular rehabilitation therapy such as balance training, acupuncture, massage, etc.

#### 2.6.3. Primary outcome

Based on the references of existing studies, the following indicators were identified as outcome indicators for judging the falling behavior of the elderly in conjunction with the “China expert consensus on falls risk assessment for the elderly (Draft)”. (1) Timed up and go test (TUGT), in which the subject stood up from the seat, walked forward for 3 m, then turned around and walked back to the chair to sit down, recording the time taken, the less time taken, the better the mobility (10). (2) Eyes closed and single legged standing balance capacity (ECLSB), the ECLSB test is performed by instructing the elderly person to stand naturally, lift either foot when the start command is heard and start timing, and stop the test when the supporting foot moves or the lifted foot hits the ground (11). (3) Berg Balance Scale (BBS), with higher scores indicating better balance (12). (4) The Modified Falls Efficacy Scale (MFES), which is a 14-item instrument that measures the fear of falling among the elderly, and the higher the total score, the more confident the participant is in his or her ability to prevent falls, i.e., good fall efficacy (13).

#### 2.6.4. Exclusion criteria

(1) Non-randomized Controlled Trials; (2) Repeatedly published Studies; (3) Non-Chinese and English Studies; (4) Trials with a large number of patients dropping out midway; (5) Research that the standard of curative effect is not clear between the test group and the control group; (6) Studies in which the test group was a non-Chinese traditional exercise therapy (Tai Chi, Ba Duan Jin, Yi Jin Jing, Wu Qin Xi).

### 2.7. Literature retrieval strategy

Published Randomized Controlled Trials (RCT) on the prevention of falls in the elderly were screened by the authors through a search of CNKI, Web of Science, PubMed, EMBASE, the Cochrane Library, and other reputable databases, all of which were searched The search period was from the establishment of the database to October 2022. A combination of subject terms and free terms were used and adapted to the characteristics of the different databases.

Chinese terms include: elderly, sports, falling, Tai Chi, Ba Duan Jin, Yi Jin Jing, Wu Qin Xi, randomized controlled trial, etc.

English terms include: (exercise OR physical activity OR sports OR training) AND (older OR elderly OR aged) AND fall^*^AND (RCT OR randomized controlled trial OR trial).

### 2.8. Literature screening and data extraction

Two separate researchers independently conducted the literature screening and data extraction. Disputes were addressed through third-party discussion and then cross-checked for accuracy. The retrieved data included: (1) basic information about the listed research; (2) basic characteristics of the subjects and their treatments; (3) risk of evaluation bias; and (4) primary outcome and measurement data.

### 2.9. Statistical analysis

The different fall behavior interventions were analyzed using R and ADDIS software, while the network relationships of the interventions were mapped. The diameter of the circle denotes the sample size of the intervention, while the width of the line between the circles represents the number of studies comparing the two therapies. Using the R-Network package and the Markov Chain Monte Carlo (MCMC) Monte Carlo approach, Network Meta-analysis and forest mapping were executed (14).

The χ^2^ test and *I*^2^ values were used to determine the magnitude of heterogeneity in the studies using ADDIS software. When *I*^2^ ≤ 50%, heterogeneity was low; when *I*^2^ = >50%~70%, heterogeneity was moderate; and when *I*^2^ > 70%, heterogeneity was high. The continuous variables were examined using the Standardized Mean Difference (SMD) as the effect statistic and the 95% Confidence Interval (CI) as the benchmark for the effect statistic.

If there was no significant difference (*P* > 0.05) across sub-groups, the Node-Split Model was used for the heterogeneity test, and the Consistency Model was used for analysis; otherwise, the Inconsistency Model was used for analysis.

ADDIS was subsequently evaluated for model convergence by using the potential scale reduced factor (PSRF) (13). If the PSRF is between 1.0 and 1.05, the convergence performance of the model is excellent, indicating that the model is stable; if the PSRF is not close to 1, the number of operations needs to be continued to be superimposed (15). Finally, the different interventions were ranked and the Chinese traditional exercise therapy was found to be the most effective in preventing and improving the falling behavior of the elderly.

## 3. Results

### 3.1. Literature retrieval results

**Figure 1:**
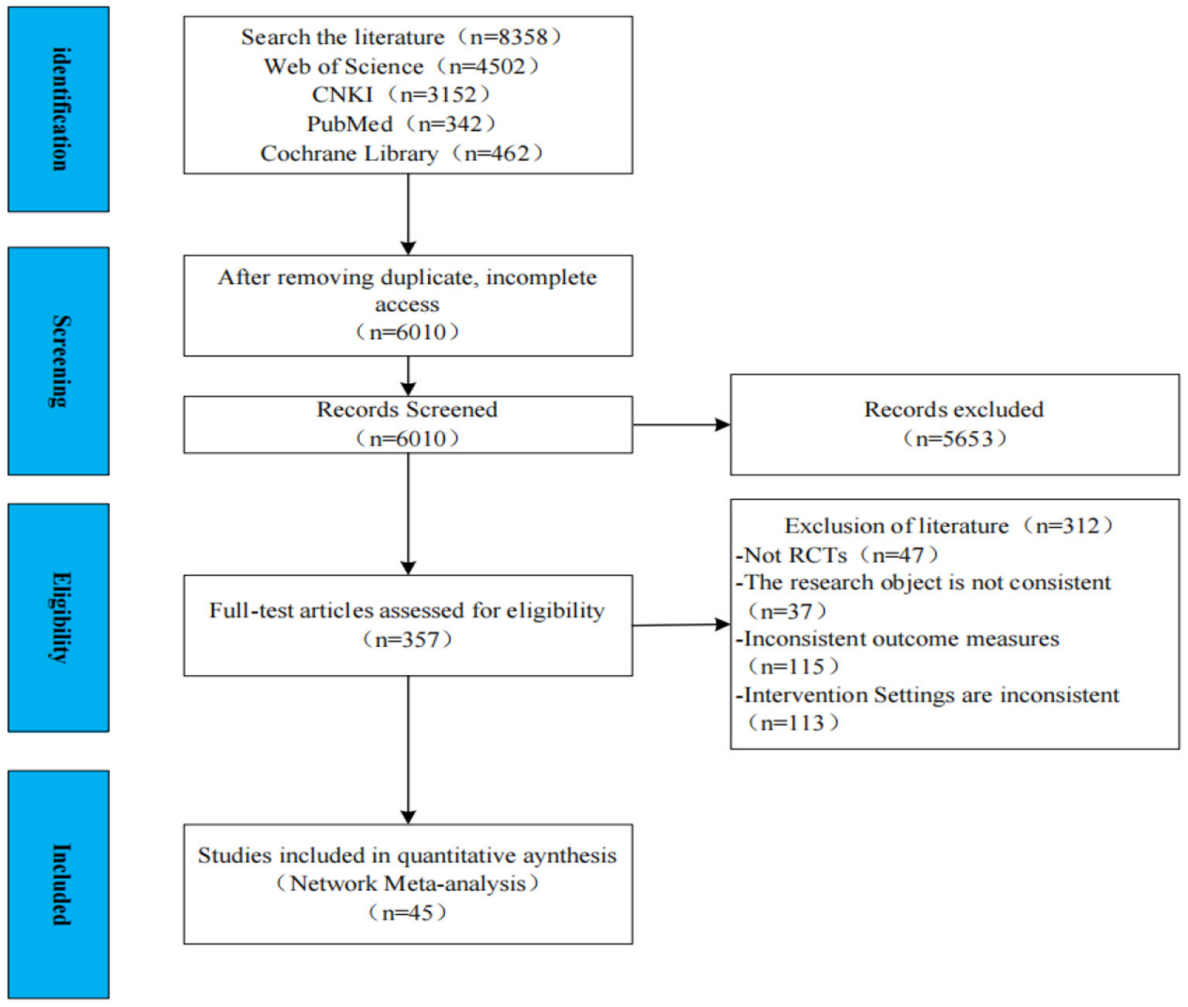
Flow chart of literature screening.

Following stratification screening, 45 RCTs involving 3,695 patients were included (Table A1). Figure 1 illustrates the literature screening method.

### 3.2. Included literature quality evaluation

Using Review Manager 5.3 software, and using the Cochrance Collaboration Network's recommended risk of bias assessment tool for literature quality evaluation. The literature quality evaluation was carried out independently by two subject members who were proficient in operating the software and finally cross-checked, with a third researcher making the judgment if disagreement was encountered. Last but not least, papers with quality A (low risk of bias) and B (moderate risk of bias) were retained, whilst those with quality C (high risk of bias) were removed.

### 3.3. Network-relational graph

**Figure 2:**
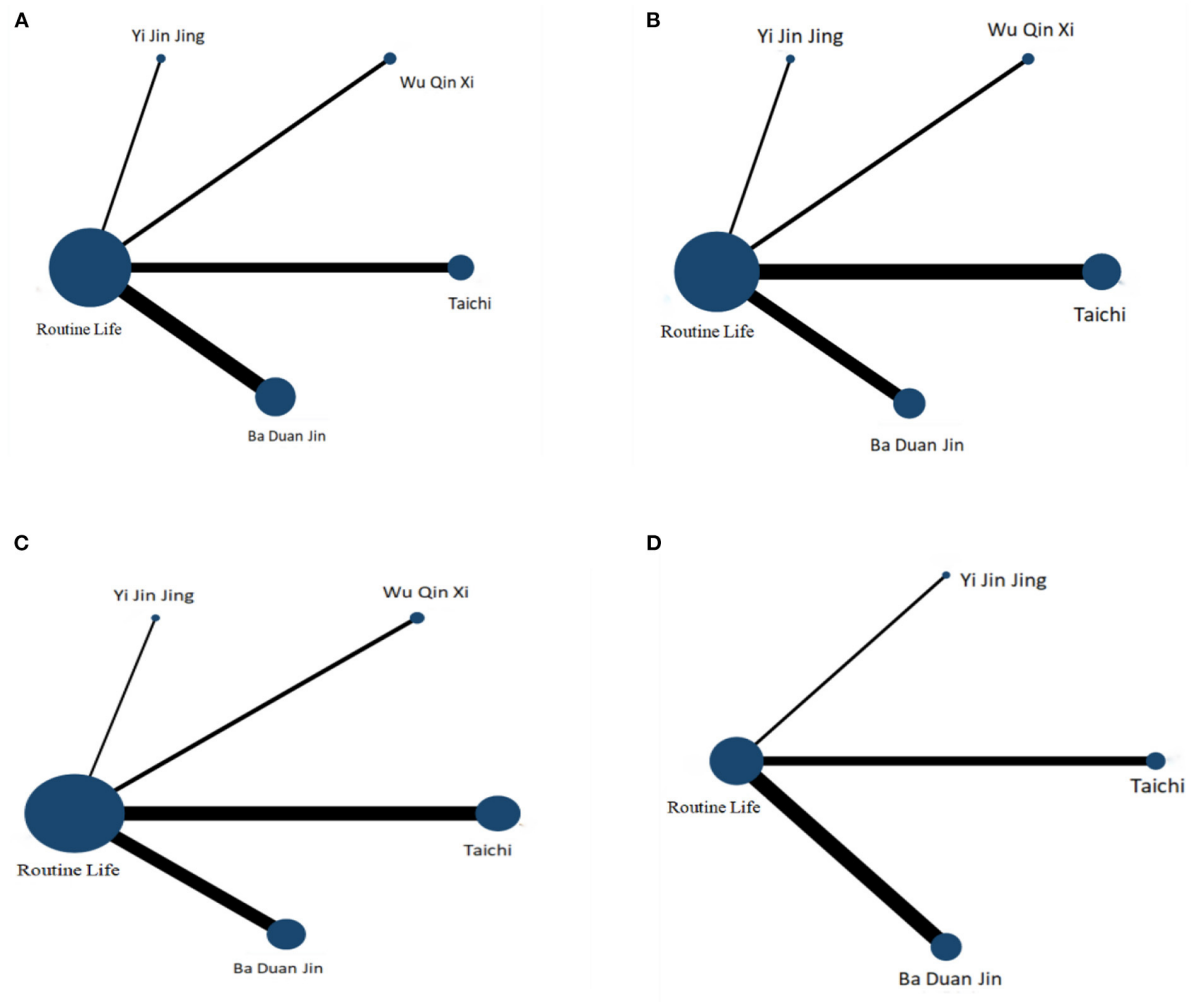
Network-relational graph of distinct motion modes under varied primary outcome. **(A)** TUGT; **(B)** ECLSB; **(C)** BBS; **(D)** MFES.

Figure 2 depicts a comparison between the four Chinese traditional exercises and the control group, with a larger sample size for conventional life interventions and fewer comparative studies for different types of traditional Chinese exercises.

### 3.4. Network meta-analysis results

#### 3.4.1. TUGT

**Figure 3:**
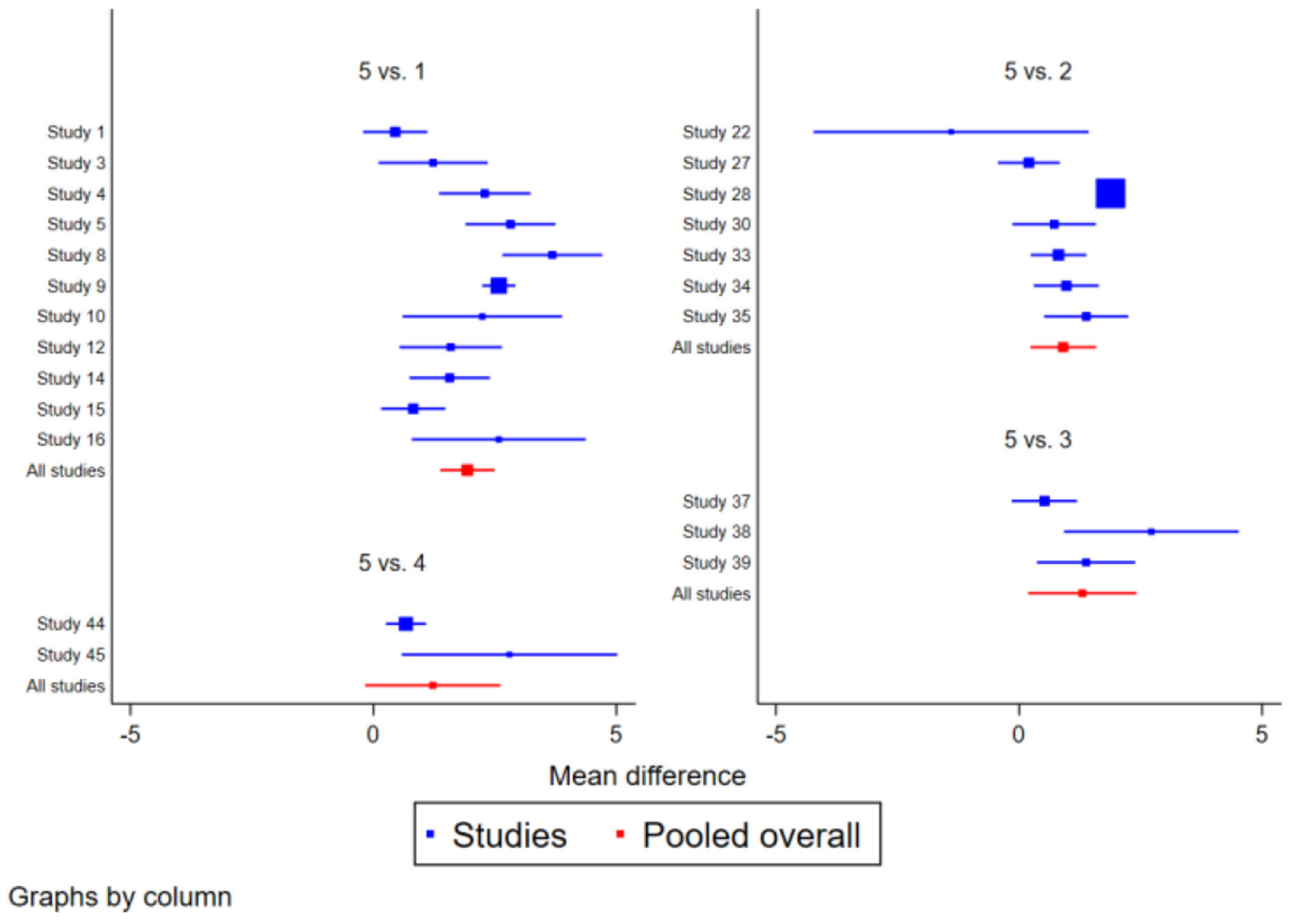
Forest plot of the effect of traditional Chinese physical exercise therapy on TUGT of the elderly.

The TUGT study included 23 studies involving 1,724 subjects. There was moderate heterogeneity (*I*^2^>50%) in the study of Ba Duan Jin, and the results were more consistent across the other methods compared. After a two-by-two comparison of the 23 studies, the Network Meta-results showed that the exercise effects of Ba Duan Jin, Tai Jin Quan, Wu Qin Xi, and Yi Jin Jing were better than those of the control group, among them, the four traditional regimes of Ba Duan Jin [SMD = −1.93 (−2.49,−1.38)], Tai Jin Quan [SMD = −0.91 (−1.58,−0.23)], Wu Qin Xi [SMD = −1.30 (−2.41,−0.19)] and Yi Jin Jing [SMD = −1.22 (−2.62,−0.17)] all effectively improved the TUGT function of the elderly, with statistically significant differences (*P* < 0.05). When the four interventions were compared in pairs, Ba Duan Jin was significantly better than Tai Jin Quan, Wu Qin Xi and Yi Jin Jing (*P* < 0.05). while there was no significant difference in other traditional items (*P* > 0.05). See Table 1 and Figure 3.

**Table 1:** Results of the network meta-analysis (SMD vs. 95% CI) of the effects of different traditional exercises on TUGT in the elderly.

Ba Duan Jin			
−1.02 (−1.90, −0.15)^*^	Tai Chi		
−0.63 (−1.87, 0.61)^*^	0.39 (−0.91, 1.70)	Wu Qin Xi	
−0.71 (−2.21, 0.79)^*^	0.31 (−1.25, 1.87)	−0.08 (−1.85, 1.69)	Yi Jin Jing
−1.93 (−2.49, −1.38)^*^	−0.91 (−1.58, −0.23)^*^	−1.30 (−2.41, −0.19)^*^	−1.22 (−2.62, 0.17)^*^

* *P* < 0.05.

**Figure 4:**
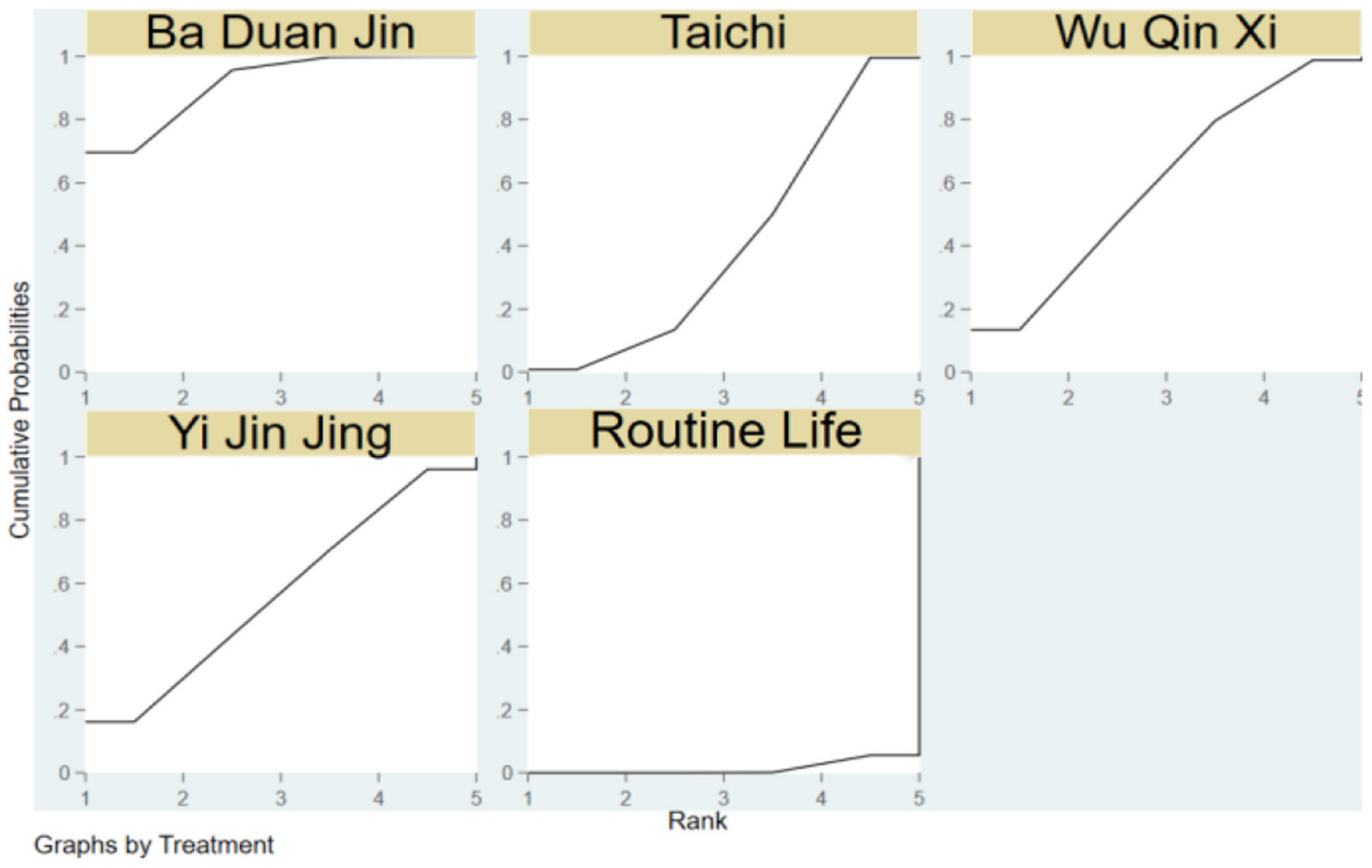
Cumulative probability SUCRA plots of the effects of different traditional programs on TUGT in the elderly.

The SUCRA of the four traditional programs in improving TUGT in the elderly is shown in Figure 4, specifically ranked as Ba Duan Jin>Wu Qin Xi>Yi Jin Jing>Tai Chi>Routine Life.

#### 3.4.2. ECLSB

**Figure 5:**
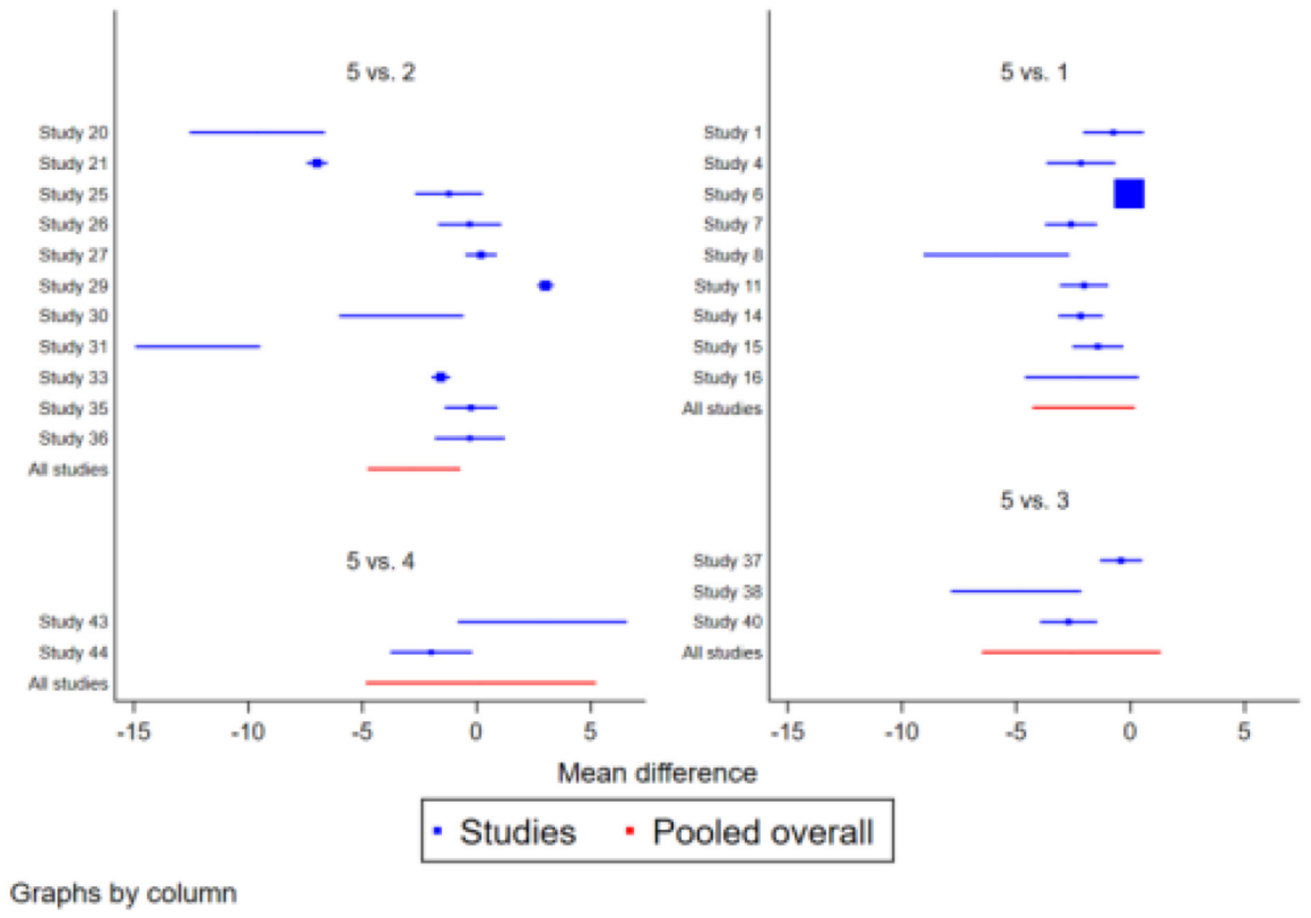
Forest map of the effect of traditional Chinese physical exercise therapy on ECLSB of the elderly.

A total of 25 studies containing 2,269 subjects were included regarding ECLSB and the results were more consistent across the other methods compared. After comparing 25 studies in pairs, the Network Meta-results showed that the exercise effects of Ba Duan Jin, Tai Chi, Wu Qin Xi and Yi Jin Jing were better than those of the control group, among them, the four traditional items of Ba Duan Jin [SMD = 2.05 (−0.14, 4.25)], Tai Chi [SMD = 2.74 (0.75, 4.73)], Wu Qin Xi [SMD = 2.59 (−1.24, 6.42)] and Yi Jin Jing [SMD = −0.19 (−5.12, −4.74)] were effective in improving the ECLSB function of the elderly, with statistically significant differences (*P* < 0.05). Compared with the 4 intervention measures, Yi Jin Jing was significantly better than Ba Duan Jin, Tai Chi and Wu Qin Xi and the difference was statistically significant (*P* < 0.05). However, there was no statistical significance among Ba Duan Jin, Tai Chi and Wu Qin Xi (*P* > 0.05). See Table 2 and Figure 5.

**Table 2:** Results of the network meta-analysis (SMD vs. 95% CI) of the effects of different traditional exercises on ECLSB in the elderly.

Ba Duan Jin			
−0.68 (−3.64, 2.28)	Tai Chi		
−0.53 (−4.95, 3.88)	0.15 (−4.17, 4.46)	Wu Qin Xi	
2.24 (−3.15, 7.64)	2.93 (−2.40, 8.25)	2.78 (−3.47, 9.03)	Yi Jin Jing
2.05 (−0.14, 4.25)	2.74 (0.75, 4.73)	2.59 (−1.24, 6.42)	−0.19 (−5.12, 4.74)^*^

* *P* < 0.05.

**Figure 6:**
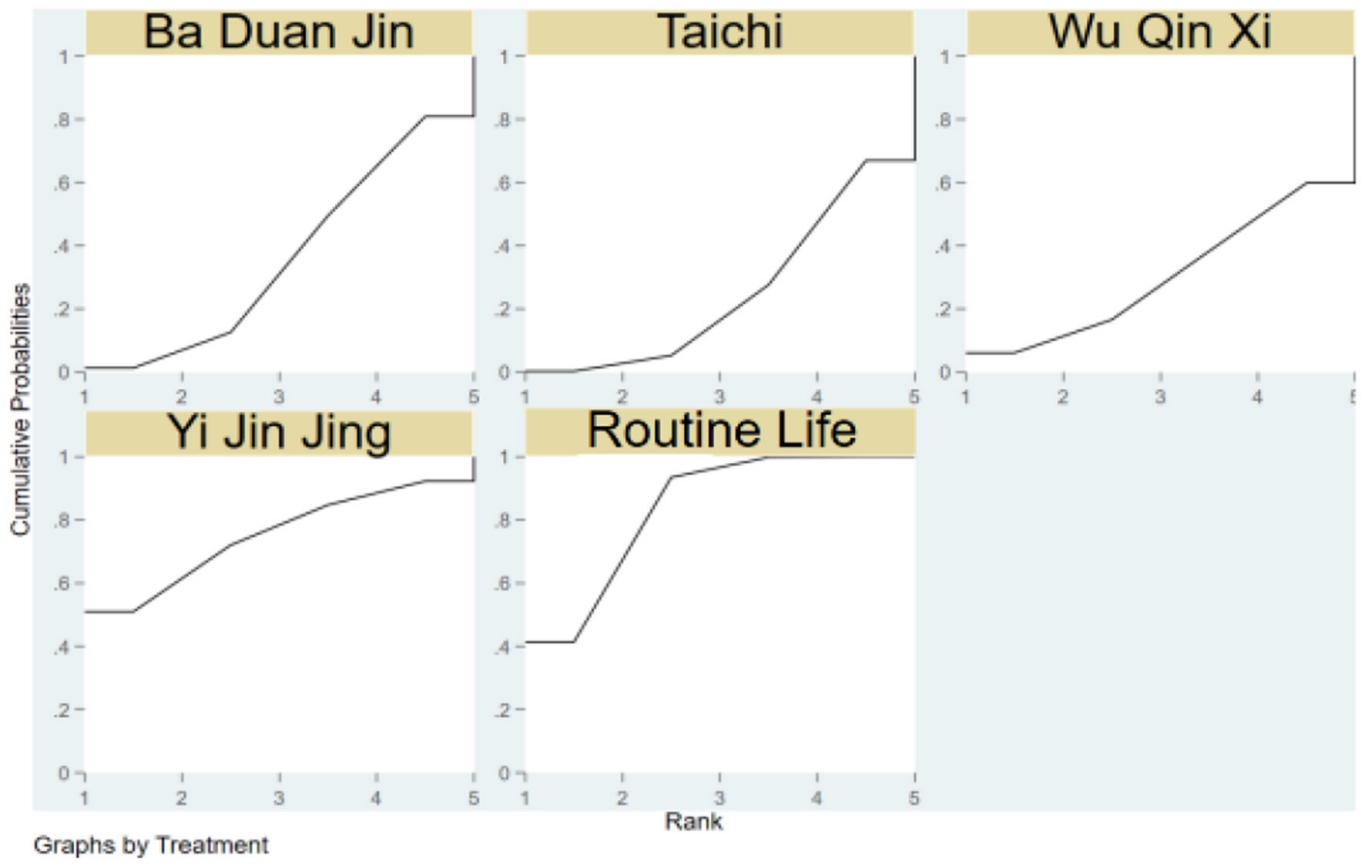
SUCRA plots demonstrating cumulative likelihood of impact of traditional items on elderly ECLSB.

Yi Jin Jing>Ba Duan Jin>Wu Qin Xi>Tai Chi>Routine Life is the SUCRA of the four traditional things for enhancing the ECLSB of the elderly, as seen in Figure 6.

#### 3.4.3. BBS

**Figure 7:**
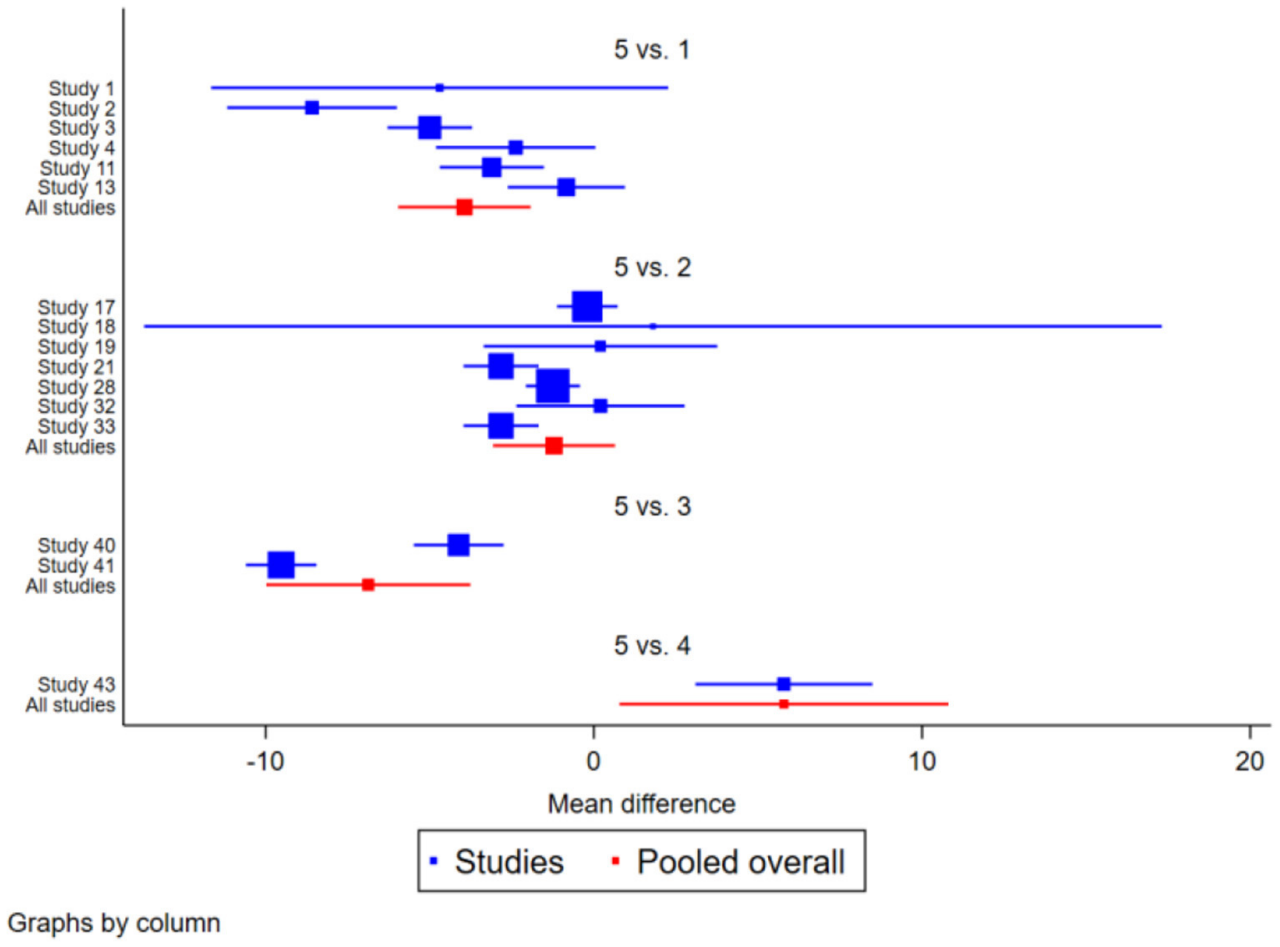
Forest plot of the effect of traditional Chinese physical exercise therapy on BBS in the elderly.

The BBS research pooled data from 16 trials, including 1,703 participants. There was moderate heterogeneity between Wu Qin Xi and Yi Jin Jing (*I*^2^ > 50%), and the results were more consistent across the other methods compared. After comparing 16 studies in pairs, the Network Meta-results showed that the exercise effect of Ba Duan Jin, Tai Chi and Yi Jin Jing was better than that of the control group, among them, the three traditional regimes of Ba Duan Jin [SMD = 3.94 (1.93, 5.96)], Tai Chi [SMD = 1.22 (−0.64, 3.08)] and Yi Jin and Jing [ SMD = −5.79 (−10.80,−0.78)] were all effective in improving the BBS function of the elderly, and the difference was statistically significant (*P* < 0.05), however, Wu Qin Xi [SMD = 6.87 (3.77, 9.98)] did not significantly improve BBS of the elderly. The effect of Yi Jin Jing was better than Tai Chi, Wu Qin Xi and Yi Jin Jing, and the effect of Ba Duan Jin and Tai Chi was better than that of Wu Qin Xi (*P* < 0.05). See Table 3 and Figure 7.

**Table 3:** Results of the network meta-analysis (SMD vs. 95% CI) of the effects of different traditional exercises on BBS in the elderly.

Ba Duan Jin			
2.73 (−0.02, 5.47)	Tai Chi		
−2.93 (−6.64, 0.78)^*^	−5.66 (−9.28, −2.04)^*^	Wu Qin Xi	
9.73 (4.33, 15.13)^*^	7.00 (1.66, 12.35)^*^	12.66 (6.76, 18.56)^*^	Yi Jin Jing
3.94 (1.93, 5.96)	1.22 (−0.64, 3.08)	6.87 (3.77, 9.98)^*^	−5.79 (−10.80, −0.78)^*^

* *P* < 0.05.

**Figure 8:**
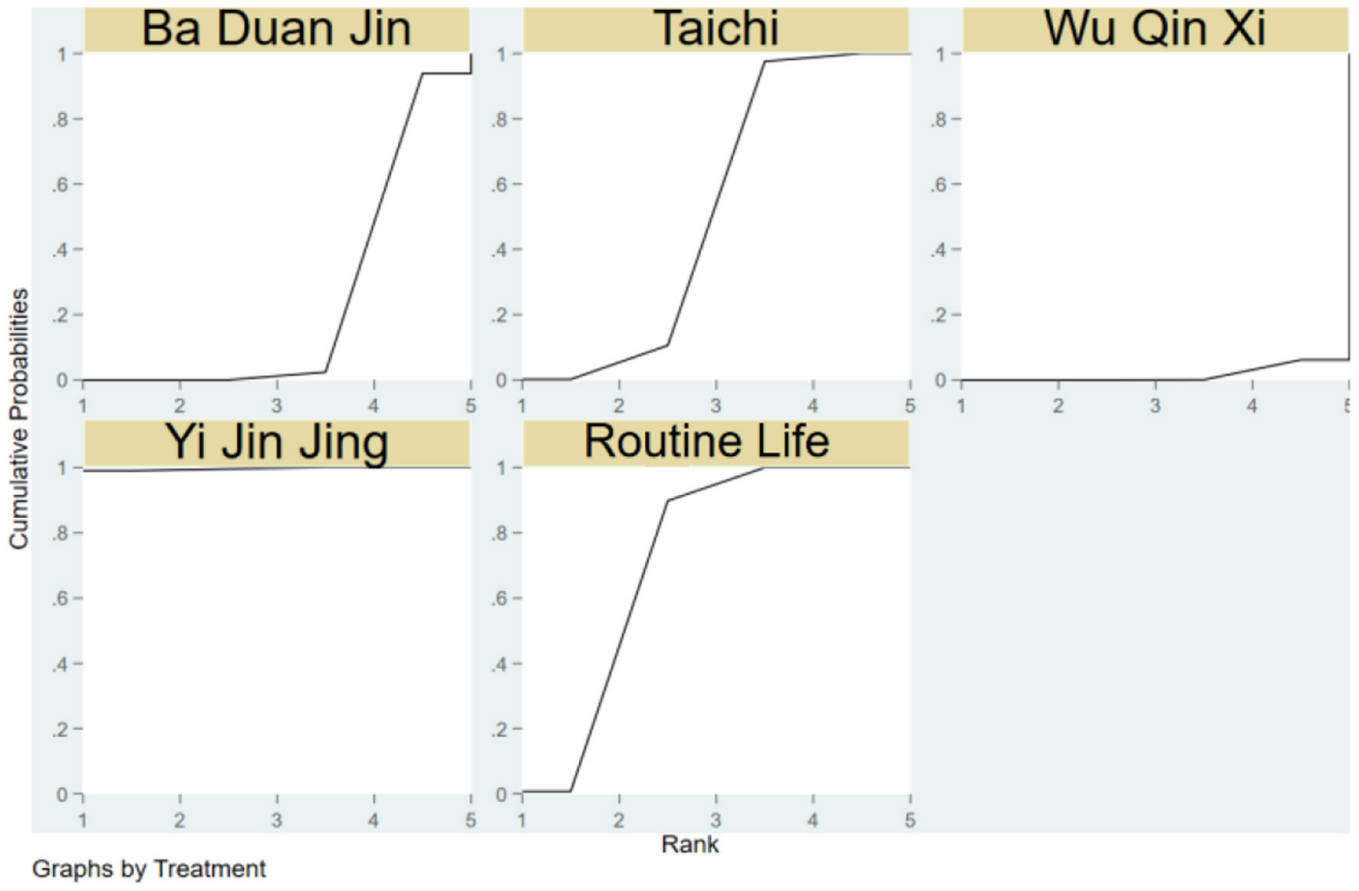
Cumulative probability SUCRA plots of the effects of different traditional programs on BBS in older adults.

The SUCRA of the four traditional programs in improving BBS in the elderly is shown in Figure 8, specifically ranked Yi Jin Jing>Tai Chi Quan>Ba Duan Jin>Routine life>Wu Qin Xi.

#### 3.4.4. MFES

**Figure 9:**
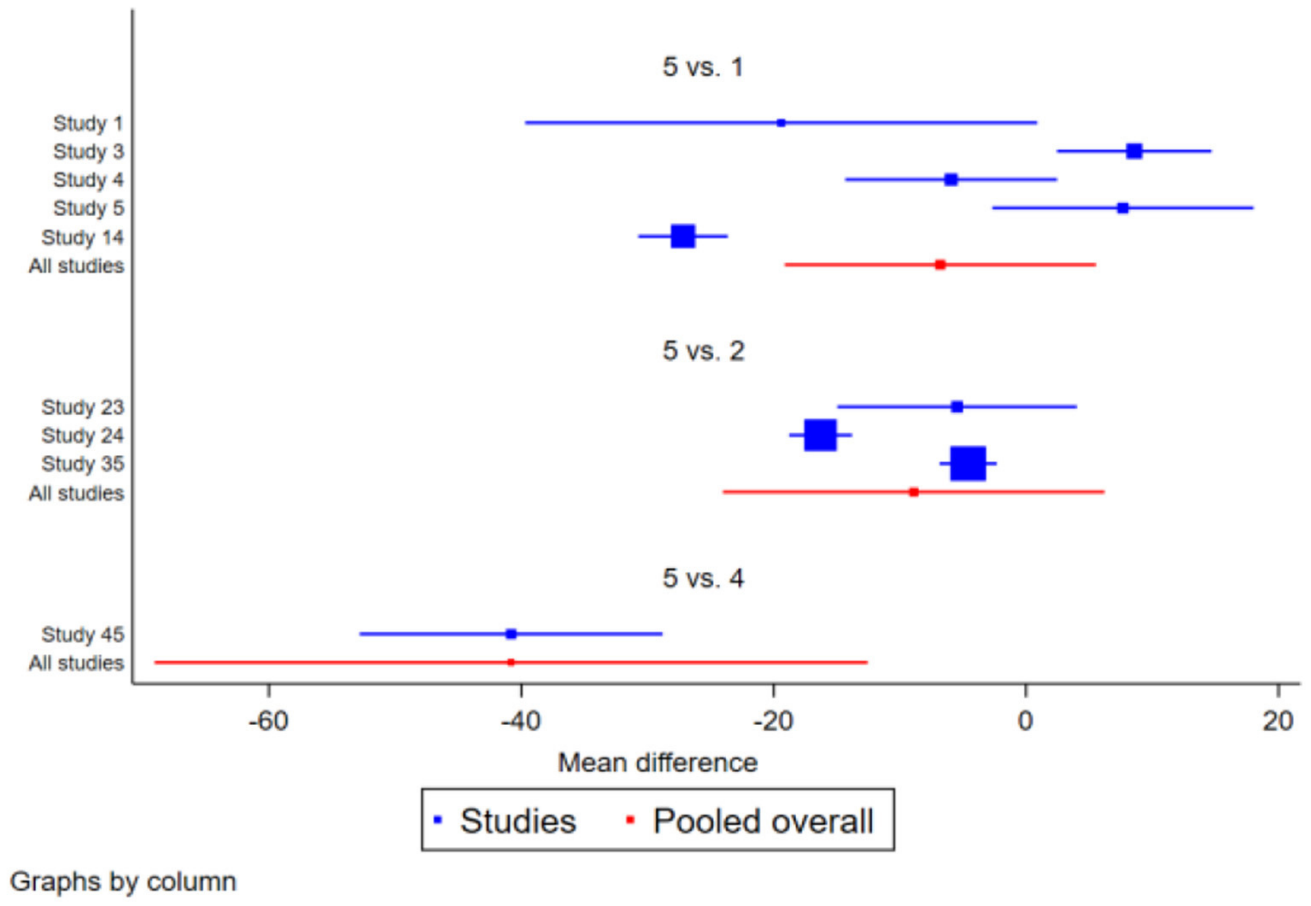
Forest plot of the effect of traditional Chinese exercise treatment on MFES in the elderly.

In the MFES analysis, a total of 9 trials comprising 451 people were considered. High heterogeneity (*I*^2^>50%) was seen in Yi Jin Jing, and the findings of other research were congruent. After comparing 9 studies in pairs, the Network Meta results demonstrated that the exercise effect of Ba Duan Jin and Tai Chi was superior to that of Yi Jin Jing. Specifically, Ba Duan Jin [SMD = −33.45 (−63.93, −2.97)] and Tai Chi [SMD = −31.15 (−63.26, 0.25)] were both effective in improving the MFES of the elderly, with the difference being statistically significant (*P* < 0.05). But the effect of Yi Jin Jing [SMD = 40.40 (12.39, 68.40)] on MFES in the elderly was not significantly improved. In contrast, the effect of routine life intervention was better than that of Yi Jin Jing. Compared with the three interventions, Ba Duan Jin and Tai Chi were significantly better than Tai Chi (*P* < 0.05). See Table 4 and Figure 9.

**Table 4:** Results of the network meta-analysis (SMD vs. 95% CI) of the effects of different traditional exercises on MFES in the elderly.

Ba Duan Jin			
−1.95 (−21.18, 17.29)	Tai Chi		
−33.45 (−63.93, −2.97)^*^	−31.51 (−63.26, 0.25)^*^	Yi Jin Jing	
6.95 (−5.22, 19.11)^*^	8.89 (−6.10, 23.88)^*^	40.40 (12.39, 68.40)^*^	Routine Life

* *P* < 0.05.

**Figure 10:**
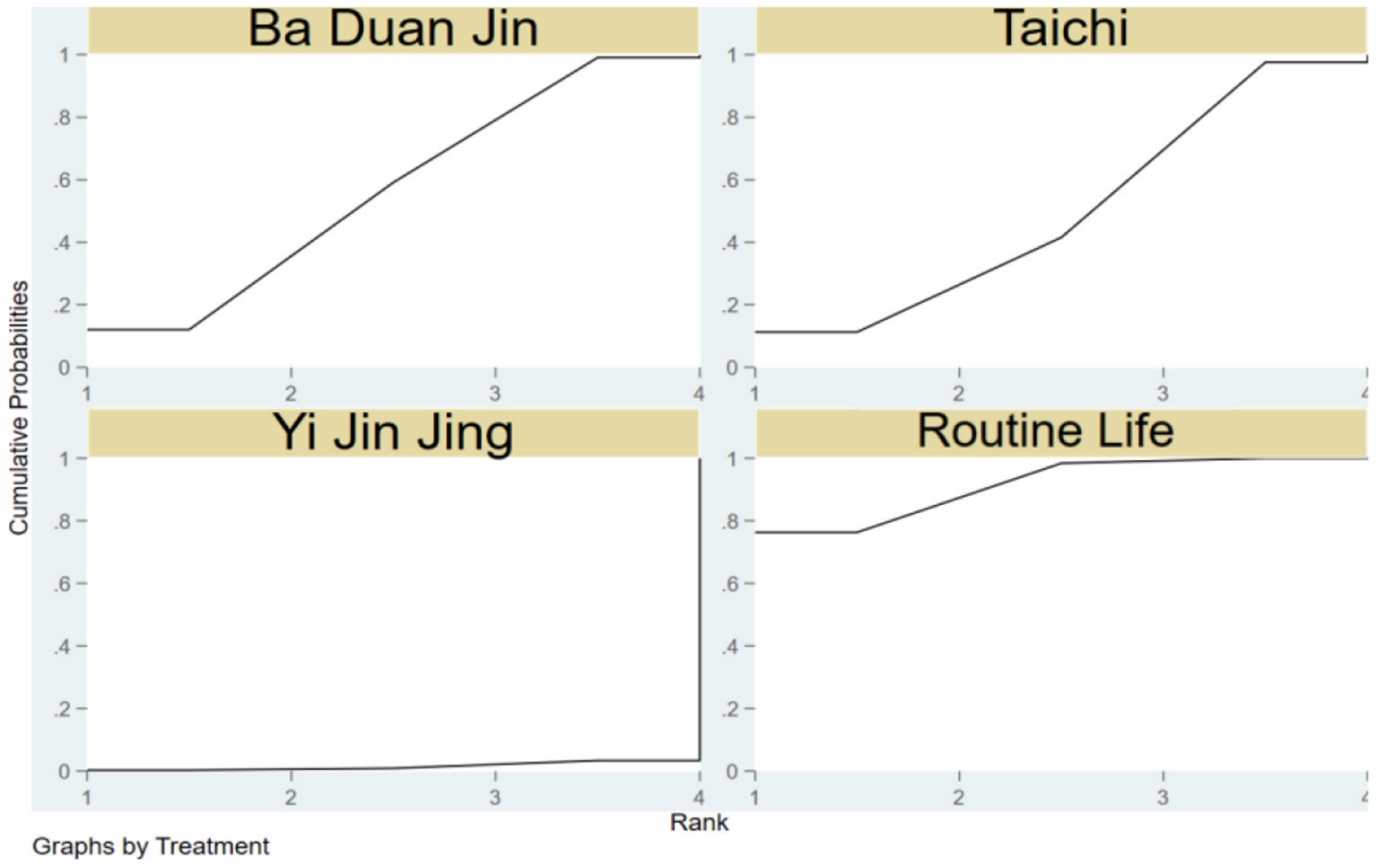
Cumulative probability SUCRA plots of the effects of different traditional programs on MFES in older adults.

Figure 10 depicts the SUCRA of the three traditional items in strengthening the MFES of the elderly, which is rated as Ba Duan Jin>Tai Chi>Routine Life>Yi Jin Jing.

### 3.5. Publication bias analysis

**Figure 11:**
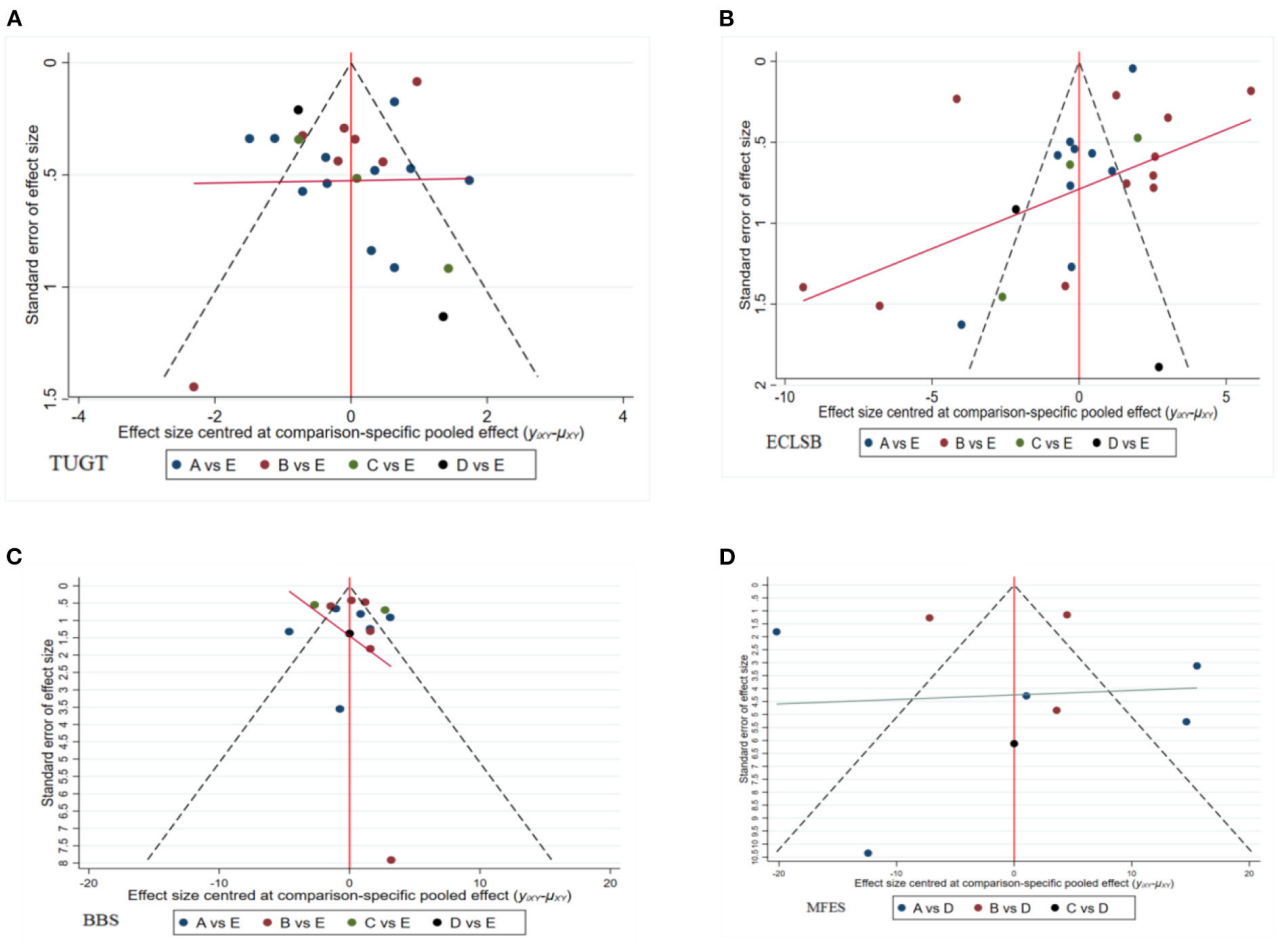
Publication bias of four Chinese traditional exercises on TUGT, ECLSB, BBS, MFES. **(A)** TUGT; **(B)** ECLSB; **(C)** BBS; **(D)** MFES. In the picture, A is Ba Duan Jin, B is Tai Chi, C is Wu Qin Xi, D is Yi Jin Jing, and E is routine life.

Of the final 45 included, 23 described the effect of traditional physical activity programs on TUGT on fall prevention behaviors in older adults, 25 described the effect on ECLSB, 16 described the effect on BBS in older adults, and 9 studies described changes in the effect on MFES in older adults. All 45 described explicitly allocation concealment, blinding, outcome data completeness, selective reporting, and follow-up. In addition, the literature was analyzed for publication bias and funnel plots were produced. Using Bgger's test for publication bias, and found that the indicators included in each study were arranged in a roughly funnel-like pattern, all distributed on either side of the baseline, with Bgger's tests showing that TUGT (Figure 11A), ECLSB (Figure 11B), BBS (Figure 11C) and MFES (Figure 11D) at *P* > 0.05, with no publication bias (Figure 11).

Figure 11 Traditional Sports Publication Bias Toward TUGT, ECLSB, BBS, and MFES.

## 4. Discussion

In recent years, traditional Chinese exercises have been widely used in preventing falls among the elderly. Four traditional Chinese exercises, namely Ba Duan Jin, Tai Chi, Yi Jin Jing and Wu Qin Xi, have become important ways of non-drug therapy. Traditional Chinese exercise provides a treatment idea and method that is different from Western medicine, that is different from Western medicine, that is, non-antagonistic and non-medical treatment methods. It treats the human body as a whole, a method of physical therapy based on reconciliation. Dialectical treatment, external and exterior complimentary, Yin-Yang, adjust Xu and Shi; and the regulation of deficiency and reality are precisely the transplanted references and subtleties of the traditional Chinese exercises method of draining and clearing the ligaments and dialectical treatment (16); Traditional Chinese medicine,” The concept of the whole, “Yin-Yang”, “Regimen”, “Meridian Theory,” and the Chinese philosophy are all integrated with the “Internal and external treatment” method, which emphasizes the use of Chinese traditional body-building exercises to prevent falls among the elderly.

This study found that the 4 Chinese traditional exercises can effectively enhance lower limb muscle strength, improve the balance of the elderly, and reduce the risk of falls (17–19). In terms of lower limb strength, especially quadriceps muscle strength decreases the strength of ligaments and tendons around the knee joint and joint stability, resulting in decreased balance function and increased risk of falls in the elderly.

According to these findings, Yi Jin Jing is the most effective treatment for enhancing ECLSB and BBS in the elderly, which is due to the effects of “Tendons are simple to strengthen, and the weak are prone to becoming strong.” as a guiding principle in the Yi Jin Jing, a traditional system of guiding methods. Through the static stretching movement of muscles and the synergistic movement of multiple joints, it can stretch the tendons and bones, stretch the muscles and improve micro-circulation and muscle mobility (17, 18). Some research has shown that Yi Jin Jing can successfully raise bone mineral density in vertebrae, increase blood flow of bone cortex, improve muscular strength and coordination, and effectively decrease the incidence of falls in the elderly (20). Other studies have shown that practicing Yi Jin Jing can improve the amount and efficiency of work done by the slow and fast muscle fibers of the body, improve skeletal muscle contraction function and coordination, and increase joint stability (19). At the same time, it has been pointed out that Yi Jin Jing, as a kind of aerobic exercise, can promote the secretion of neurotransmitters (such as serotonin), which helps the emotional stability of the elderly and thus relieves the psychological stress of the practitioners (21, 22). Accordingly, there is promising evidence that Yi Jin Jing may improve senior people's muscular strength, joint stability, and psychological stress levels; nevertheless, the underlying molecular mechanism of fall prevention still has to be investigated in depth.

The findings demonstrated that Ba Duan Jin had a favorable effect on the TUGT and MFES of the elderly. Ba Duan Jin is the most notable and significant ancient Chinese technique Among the traditional Chinese Gong Fu methods, Ba Duan Jin is one of the most widespread and influential forms of exercise, which is popular among elderly practitioners because of its long history, various forms, simplicity and effectiveness. Multiple studies have shown that older persons who practice Ba Duan Jin are able to thoroughly condition the forearm and hand muscle groups, delay cognitive decline, improve neurohumoral regulation, and prevent and improve age-related diseases such as arthritis and osteoporosis (23, 24). The influence of this study on the functional evaluation and increase of physical activity among Ba Duan Jin practitioners is equivalent to that of previous research. Due to Ba Duan Jin's training, the TUGT time was decreased and the MFES score was significantly increased. As a result, the likelihood of falling behavior among the elderly decreased. Frequent, low-intensity physical exercise lowers the frequency of falls among the elderly, as shown by Morgan et al. (25) finding is consistent with the present study, as Ba Duan Jin is a low-intensity form of physical exercise suitable for the elderly, and through long-term and regular practice, it can improve the physiological function of the elderly, thus helping to reduce the risk of falls.

This investigation has certain limitations. Some of the included literature did not report on blinding, and there were significant differences in the physical condition, age differences, trial format and trial period of the study participants, resulting in a large amount of heterogeneity in the study, which may cause bias in the findings. Additionally, the study only examined the impact of four Chinese traditional exercise techniques (Tai Chi, Ba Duan Jin, Yi Jin Jing, and Wu Qin Xi) on reducing the risk of falls in the elderly; owing to this, other body-building techniques (Six Letters Formula, Ba Duan Jin, 12-Step Daoyin Health Preservation Exercises, 12 Routine Exercises, Dawu Exercises, Taiji Stick Health Preservation Exercises, etc.) were not included in the study. There should be more well-designed research investigated in future studies.

In future studies, research trials should be designed in a more objective and standardized manner, and high-quality studies such as prospective, large sample and double-blind randomized controlled trials should be selected for further verification as much as possible, so as to reduce the risk of research bias to the greatest extent and scientifically screen out the Chinese traditional therapy schemes with better clinical effects.

## 5. Conclusion

The findings of the Network Meta-analysis indicated that the four Chinese traditional exercises had beneficial preventative and therapeutic effects on falls in the elderly, which was consistent with the 45 included studies. Among them, Ba Duan Jin has more preventative and therapeutic benefits in boosting TUGT and MFES, while Yi Jin Jing has greater preventive and therapeutic effects in promoting ECLSB and BBS in the elderly. Therefore, in clinical treatment and daily physical exercise, Ba Duan Jin and Yi Jin Jing can be preferred for effective prevention and treatment of fall behavior, however, they should also be combined with the actual conditions of the patients, scientific and rational choice of exercise programme. This analysis was restricted by the quantity and quality of included papers, and the aforementioned results are yet to be verified by more high-quality studies.

## Data availability statement

The original contributions presented in the study are included in the article/Supplementary material, further inquiries can be directed to the corresponding authors.

## Author contributions

MC: conceptualization, methodology, software, formal analysis, writing—original draft, and writing—review and editing. YW: conceptualization, methodology, and software. SW: conceptualization, supervision, project administration, funding acquisition, and writing—review and editing. WC: data curation and validation. XW: supervision and thesis revision guidance. All authors have read and agreed to the published version of the manuscript.
