# Supplementary material for: Network meta-analysis of the efficacy of four traditional Chinese physical exercise therapies on the prevention of falls in the elderly
Source: Front Public Health. 2023 Jan 4;10:1096599. doi: 10.3389/fpubh.2022.1096599 (PMC9846771; doi:10.3389/fpubh.2022.1096599)
Supplement: Supplementary file 1 [file Table_1.pdf]

Appendix

Table A1. Basic Characteristics of the Included Literature.

Sequence Number	Literature Author	Published Year	Sex	Age		Sample	Test Group	Control Group	Sport Event		Hz /(time·week-1)	Period	Primary outcome
				Test Group	Control Group				Test Group	Control Group			
1	Shi Xiaoyan	2017	Male、Female	64.7±6.3	65.5±6.5	104	52	52	Ba Duan Jin	Walking Exercise	2	12	(TUGT)、(ECLSB)、（BBS）、(MFES)
2	Kuang Xiaowen	2019	-	68.68±3.22	63-79	82	41	41	Ba Duan Jin	Conventional Medicine	-	-	（BBS）
3	Wu Xia	2017	Male、Female	70.55±4.26	70.55±4.26	120	60	60	Ba Duan Jin	Routine Life	14	4	(TUGT)、（BBS）、(MFES)
4	Liu Xiaoyun	2016	Male、Female	67.1±6.2	67.1±6.2	47	27	20	Ba Duan Jin	Routine Life	2	12	(TUGT)、(ECLSB)、（BBS）、(MFES)
5	Zhou Supo	2020	Male、Female	72.67±9.56	73.25±8.54	40	20	20	Ba Duan Jin	Health Education	5	8	(TUGT)、（BBS）
6	Du Wenyuan	2018	Male、Femal	65.36±4.62	67.21±5.61	160	97	63	Ba Duan Jin	Health Education	5	24	(ECLSB)、

			e										
7	Sun Jiao	2022	Male、Female	68.3±5.9	70.1±5.7	138	72	66	Ba Duan Jin	Routine Life	2	12	(ECLSB)、
8	Xu Mengyao	2022	Male、Female	67.47±8.25	67.33±5.96	32	17	15	Ba Duan Jin	Routine Life	3	12	(TUGT)、(ECLSB)、
9	Gao Zhipeng	2020	Male、Female	79.79±4.18	78.88±4.66	68	34	34	Ba Duan Jin	Health Education	5	12	(TUGT)、
10	Hou Xiaolin	2018	Male、Female	82.86±4.37	83.94±3.38	71	36	35	Ba Duan Jin	Resistance Training	5	12	(TUGT)、
11	Er Yuliang	2017	Female	67.23±4.54	67.22±4.72	144	71	73	Ba Duan Jin	Health Education	5	12	(ECLSB)、（BBS）、
12	Liu Shuangtao	2015	—	82.14±1.68	84.15±2.95	15	7	8	Ba Duan Jin	Routine Life	4	12	(TUGT)、
13	Zhang Lifen	2012	Male、Female	64.60±2.30	65.10±1.90	60	30	30	Ba Duan Jin	Routine Life	4	24	（BBS）、
14	Zhao Lei	2021	Male、Female	65.82±3.88	64.35±3.62	34	17	17	Ba Duan Jin	Routine Life	3	12	(TUGT)、(ECLSB)、(MFES)
15	Zhou Jing	2020	Male、Female	59.70±6.23	61.13±6.04	61	30	31	Ba Duan Jin	Routine Life	5	13	(TUGT)、(ECLSB)、

16	Hou Yanshao	2014	Male、Female	60-69	60-69	40	20	20	Ba Duan Jin	Routine Life	5	12	(TUGT)、(ECLSB)、
17	IngeH.	2009	Male、Female	77.5±4.7	76.8±4.6	269	138	131	Tai Chi	Routine Life	2	13	（BBS）、
18	Padma	2014	Male、Female	81.1±8.0	85.4±9.1	22	11	11	Tai Chi	Routine Life	2	14	（BBS）、
19	Michel	2013	Male、Female	79.1±6.4	80.7±6.0	80	42	38	Tai Chi	Conventional Medicine		48	（BBS）、
20	Jian-Guo	2006	Male、Female	70.2±3.6	70.6±4.9	47	24	23	Tai Chi	Routine Life	7	8	(ECLSB)、
21	Fuzhong	2005	Male、Female	76.94±4.69	77.99±5.14	256	125	131	Tai Chi	Routine Life	3	24	(ECLSB)、（BBS）、
22	Deborah	2019	Male、Female	80.4±6.8	71.2±6.1	29	16	13	Tai Chi	Routine Life	4	16	(TUGT)、
23	류명인	2008	Male、Female	77.70±5.41	77.50±6.58	47	23	24	Tai Ji Exercise	Routine Life	2	12	(MFES)
24	Hamed	2018	Male、Femal	67.20±5.416	68.07±5.232	53	27	26	Tai Chi	Routine Life	3	10	(MFES)

			e										
25	Jung	2005	Male、Female	76.96±7.7	78.73±6.9	59	29	30	Tai Chi	Routine Life	3	12	(ECLSB)、
26	JOSEPH	2006	Female	71.5±4.6	71.3±4.4	19	11	8	Tai Chi	Jogging	3	12	(ECLSB)、
27	Denise	2012	Male、Female	74.4±6.2	73.7±6.2	451	220	231	Tai Chi	Low-level Exercise	2	24	(TUGT)、(ECLSB)、
28	Hu Xiu	2021	Male、Female	66.4 ±1.7	66.7 ±1.8	31	15	16	Tai Chi	Health Education	4	16	(TUGT)、（BBS）、
29	Hong Du	2018	Male、Female	71.26±7.95	69.97±8.03	72	37	35	Tai Chi	Health Education	3	24	(ECLSB)、
30	Yang Shoulan	2021	Male、Female	Male：65.08±4.05 Female：61.26±5.25	Male：65.08±4.05 Female：61.26±5.25	40	20	20	Tai Chi	Routine Life	-	-	(TUGT)、(ECLSB)、
31	Qiu Yuyu	2008	Male、Female	Above 65	Above 65	53	27	26	Tai Chi	Routine Life	7	10	(ECLSB)、
32	Tousignant M	2013	Male、Female	79.1±6.4	80.7±6.0	152	76	76	Tai Chi	Conventional Medicine	2	15	（BBS）、
33	Li F	2005	Male、Fe	76.94±4.69	77.99±5.14	256	125	131	Tai Chi	Routine Life	3	26	(TUGT)、(ECL

			male										SB)、（BBS）、
34	Zhao Y	2017	Male、Female	70.2±3.9	69.9±3.3	41	20	21	Tai Chi	Routine Life	3	16	(TUGT)、
35	Zhang Jinjin	2019	Male、Female	67.8±3.6	66.3±3.2	32	16	16	Tai Chi	Routine Life	3	12	(TUGT)、(ECLSB)、(MFES)
36	Wang Rui	2016	Male、Female	55.2±6.2	57.3±6.5	40	20	20	Tai Chi	Routine Life	5	16	(ECLSB)、
37	Qi Mandi	2020	Male、Female	70.57±7.41	65.13±7.34	66	32	34	Wu Qin Xi	Routine Life	5	12	(TUGT)、(ECLSB)、
38	Hou Yanshao	2014	Male、Female	60-69	60-69	40	20	20	Wu Qin Xi	Routine Life	5	12	(TUGT)、(ECLSB)
39	Guo Yadong	2018	Male、Female	65.15±3.74	63.95±6.68	40	20	20	Wu Qin Xi	Home Exercise	2	12	(TUGT)、
40	Zhang Gai	2019	Male、Female	65.06±4.905	63.94±5.068	34	17	17	Wu Qin Xi	Routine Life	2	12	(ECLSB)、BBS）、
41	He Peng	2012	Male、Female	65-70	65-70	60	30	30	Wu Qin Xi	Routine Life	3	26	（BBS）、

42	Luo Kailiang	2022	Male、Female	68.08±5.90	66.25±6.24	48	24	24	Yi Jin Jing	Conventional Medicine	5	4	（BBS）、
43	Zhang Boxin	2009	Male、Female	63±5	63±5	56	26	30	Yi Jin Jing	Routine Life	3	12	(ECLSB)、
44	Hu Li	2017	Female	65.24±2.94	65.52±3.09	50	25	25	Yi Jin Jing	Routine Life	3	16	(TUGT)、(ECLSB)、
45	Fang Lei	2020	Male、Female	60-95	60-95	36	18	18	Yi Jin Jing	Routine Life	3	24	(TUGT)、(MFES)
